# Left side perforated appendicitis with intestinal non-rotation: a case report

**DOI:** 10.1186/s13256-023-03990-2

**Published:** 2023-06-30

**Authors:** Mezgebu Alemneh Assefa, Yohannis Derbew Molla, Mensur Osman Yasin, Oumer Ahmed Ali, Zerubabel Tegegne Desita

**Affiliations:** 1 Department of Surgery, University of Gondar Specialized Hospital, Gondar, Ethiopia; 2 Department of Radiology, University of Gondar Specialized Hospital, Gondar, Ethiopia

**Keywords:** Acute appendicitis, Mal-rotation, Abdominal pain, Case report

## Abstract

**Background:**

Acute appendicitis is the most common cause of acute abdominal pain, requiring emergency surgery. Symptoms and signs of acute appendicitis usually occur in the right lower quadrant. However, approximately one-third of cases have pain unexcepted location due to its various anatomical locations. Acute appendicitis is a very rare cause of left lower quadrant pain; if it occurs, situs inversus (SI) and midgut malrotation (MM) are uncommon anatomic anomalies that complicate its diagnosis and management.

**Clinical presentation:**

Here we present a 23-year-old Ethiopian male patient who presented with epigastric and left paraumbilical abdominal pain, fever, and vomiting of a day duration. On examination at admission, the patient had left lower quadrant tenderness. Later, with the help of imaging studies, the patient was diagnosed with left-side acute perforated appendicitis with intestinal nonrotation, and he was operated on and discharged improved after 6 days of hospital stay.

**Conclusion:**

Physicians should be aware that acute appendicitis in patients with intestinal mal-rotation may be present with left-side abdominal pain. Although it is extremely rare, acute appendicitis should always be considered in the differential diagnosis of left-side abdominal pain. An increase in awareness of this anatomical variant is essential for physicians.

**Supplementary Information:**

The online version contains supplementary material available at 10.1186/s13256-023-03990-2.

## Introduction

Midgut malrotation (MM) is largely a pediatric diagnosis, but initial detection can be made in adulthood [1, 2]. It is a congenital anomaly that arises from incomplete rotation or abnormal position of the midgut during embryonic development [3]. Malrotation can present either acutely, intermittently, or asymptomatically. There are different types of Mid-gut Malrotation: non-rotation, incomplete rotation, or reverse rotation [4]. MM is a rare anatomic anomaly that complicates the diagnosis and management of acute abdominal pain. Abdominal pain due to acute appendicitis is one of the most causes of emergency presentation requiring surgical consult and treatment. Since acute appendicitis (AA) is commonly recognized clinically by migratory right iliac fossa pain, clinicians and surgeons are usually trained to diagnose and operate on a right-sided appendix, thus, diagnosing and promptly intervening on left-sided appendicitis is quite challenging. The occurrence of anatomical anomalies should be considered especially when clinical and imaging features are misleading [5]. Diagnosis of left-sided acute appendicitis (LSAA) with non-rotation of the colon is rare and usually creates a dilemma and often makes the diagnosis of a patient difficult [6]. Left-sided acute appendicitis should be considered in the differential diagnosis of patients with pain localized in the left lower quadrant. Increased awareness of this entity and an understanding of its varied presentation at different ages may reduce delays in diagnosis and improve patient outcomes [7]. Here we discussed a rare presentation of left-sided perforated appendicitis with midgut non-rotation in a 23-years-old male patient.

## Case presentation

We present a 23-years-old Ethiopian male patient who presented with epigastric and left-side abdominal pain and vomiting of 1-day duration. He has a history of low-grade fever and watery diarrhea 6–8 times per day for the same duration. He denied any history of previous abdominal surgery and medical illnesses. He denied any history of trauma to the abdomen. He was only a social drinker and had no previous history of similar illnesses. He has no history of diabetes, hypertension, or asthma.

On examination, his vital signs (blood pressure of 110/75 mmHg, temperature of 36.9 °C, pulse rate of 84 beats per minute, and respiratory rate of 14 breathes per minute) were normal and he had left lower quadrant tenderness and rebound tenderness. On investigations, laboratory tests: complete blood count (Hct of 39.4, white blood cell count of 4700 with 73% neutrophilia and platelet count of 230,000/mcl of blood), random blood sugar (79 g/dL), serum electrolytes (Na-138 meq/L) and K-3.7 meq/L), liver function tests (SGOT = 24 U/L, SGPT = 19 U/L, and Albumin = 4.3 g/dL) and renal function tests (Creatinine = 0.6 mg/dL and BUN = 23) were normal. Stool examination showed trophozoites of entamoeba histolytica and many pus cells. Therefore, with a diagnosis of amebic colitis IV metronidazole was started. Despite being managed for intestinal amebiasis, the pain and the abdominal tenderness persisted and progressively worsen. Therefore, an abdominal ultrasonography (USG) examination was ordered. Initial abdominal USG showed normal findings except for the non-visualized appendix. Subsequently, Contrast-Enhanced Computed Tomography (CT) of the abdomen was ordered and a pre-contrast CT scan revealed that the small bowel was on the right half of the abdomen and the large bowel was on the left half of the abdomen. There was also fluid and free air within the peritoneal cavity.

**Fig. 1:**
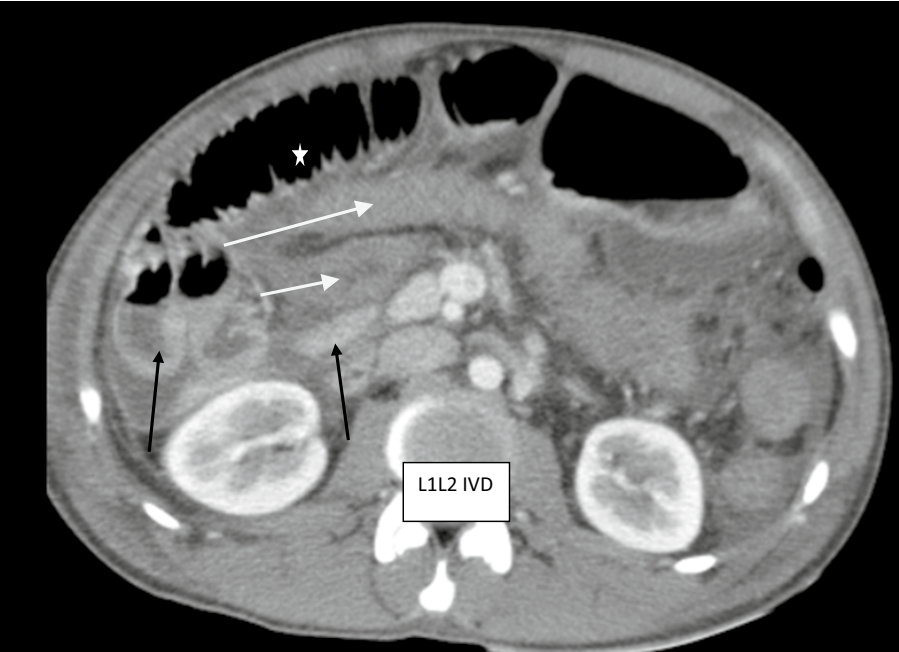
Post contrast computed tomography axial image showing transverse colon (long white arrow), posteromedial & left side directed ascending colon (white short arrow), right sided jejunum & duodenojejunal junction (black arrows) and distended small bowel anteriorly (star)

**Fig. 2:**
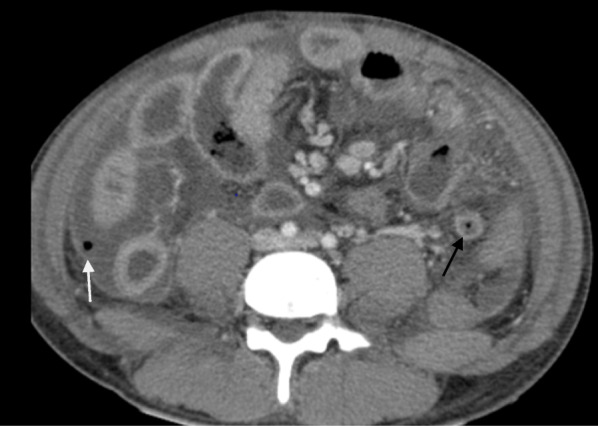
Post contrast computed tomography scan image showing left side appendicitis (black arrow) with peritoneal air & fluid (white arrow)

**Fig. 3:**
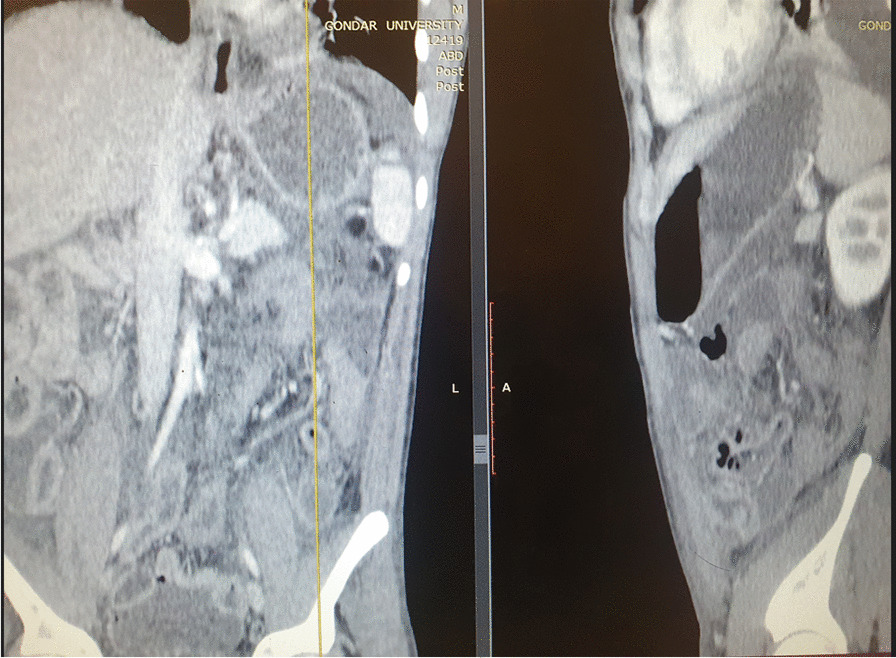
Coronal & sagittal reconstructed post contrast computed tomography scan images with left side thickened appendix

**Fig. 4:**
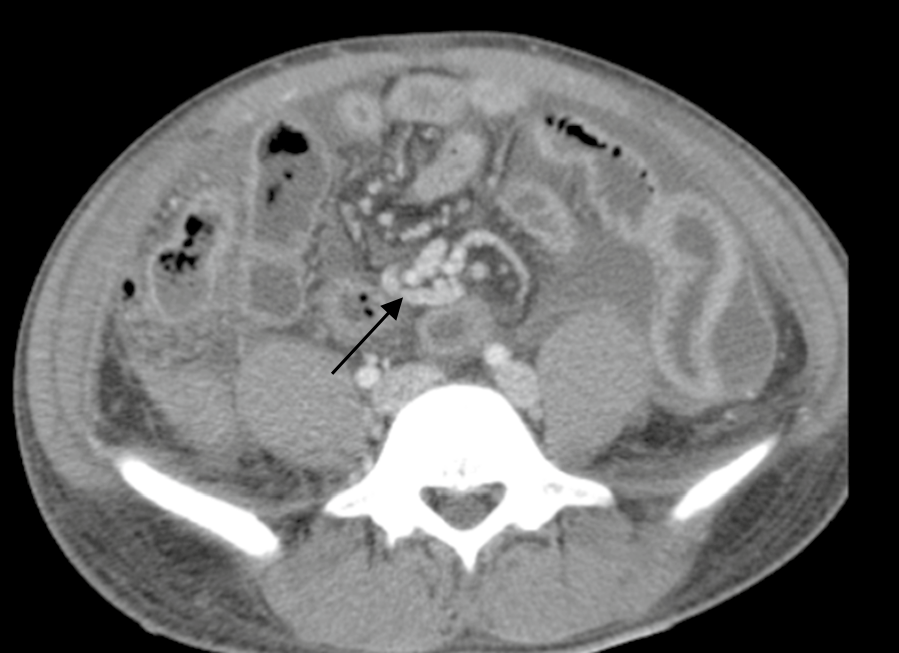
Post contrast computed tomography scan of the lower abdominal axial image showing a branch of superior mesenteric vein curling around the distal superior mesenteric artery (arrow), fluid filled small bowel loops & ascites

**Fig. 5:**
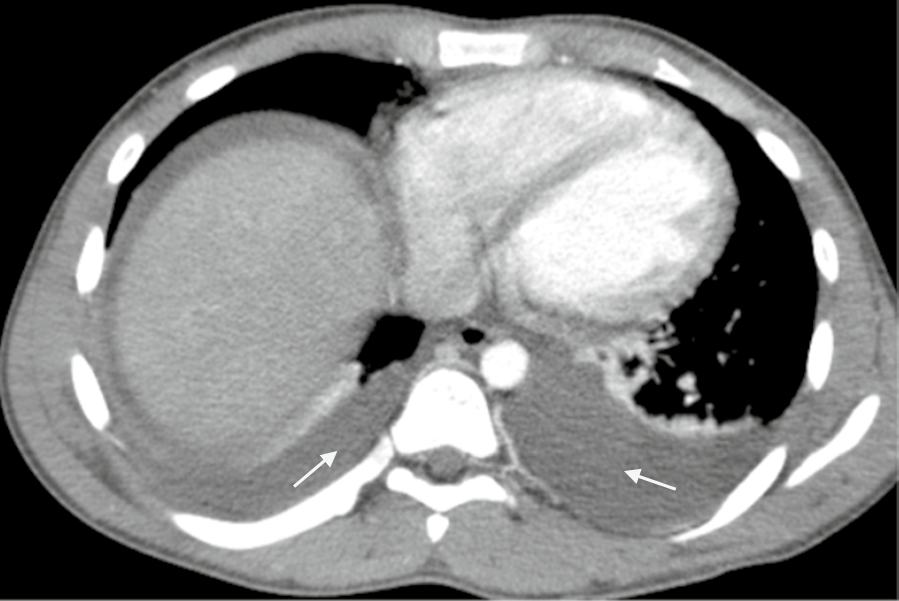
Upper abdominal & lower chest post contrast computed tomography axial image with bilateral pleural effusion (arrows)

**Fig. 6:**
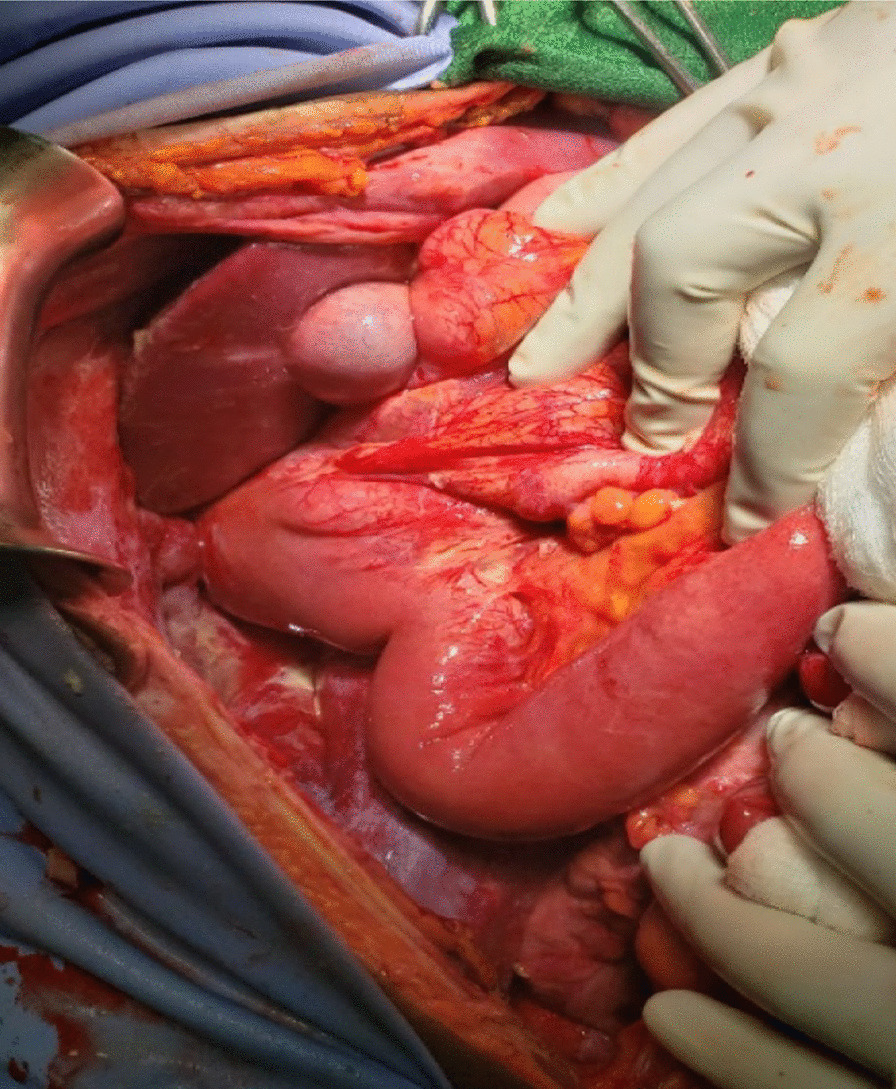
The ligament of Trietz and the small bowel shown on the right side beneath the right lobe of the liver with absent appendix, cecum and ascending colon from the right side

Post-contrast CT scan of the abdomen showed mal-rotated bowel with right-sided jejunum and the duodenojejunal junction located anterior to the L1 vertebral body just below the neck of the pancreas (Fig. 1). There was a medially located ascending and transverse colonic junction anterolateral to the L2 vertebral body with twisted ascending colon posteromedial to the transverse colon and coursed to the left lower quadrant ending at the ileocecal junction (Fig. 1). Left-sided thickened (16 mm thick) and the gas-containing appendix was seen at the left lower quadrant adjacent to the left-sided ileocecal junction (Figs. 2, 3). There was a moderate peritoneal free fluid collection with intra-peritoneal air (Fig. 2). Dilated midline located main superior mesenteric vein was seen anterior to the superior mesenteric artery but no twist around the artery. There was a branch of the superior mesenteric vein twisted around the distal superior mesenteric artery (Fig. 4). A right lobe segment V hepatic hypo-dense 4*3 cm solid mass with peripheral nodular progressive post-contrast enhancement was seen on a background of normal parenchymal texture and density (Fig. 5). There was also bilateral pleural effusion (Fig. 6).

**Fig. 7:**
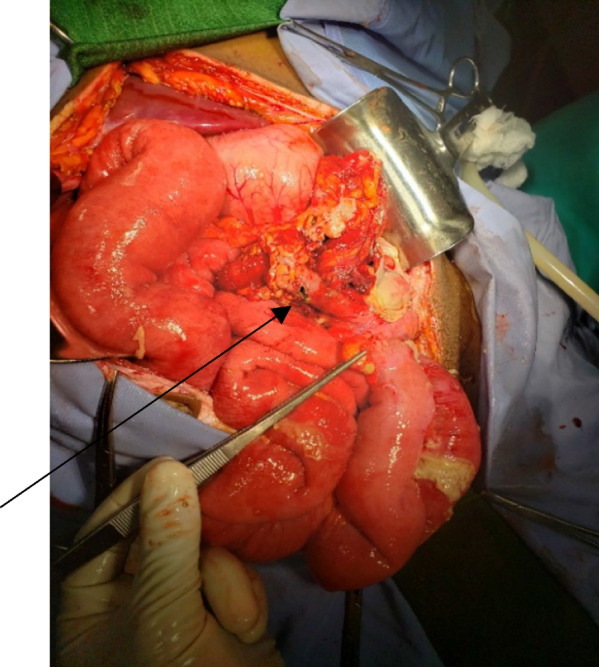
The entire colon is on the left side with perforated mid third of the appendix just inferior to the stomach (the black arrows)

The patient was on close follow-up and has been taking antibiotics for GI-onset sepsis with amebic dysentery for which he didn’t show any improvements till 48 hours after admission (Table 1). Subsequently, the patient was diagnosed with generalized peritonitis secondary to left-side perforated appendicitis and an emergent laparotomy was performed. Intraoperative surgical findings revealed that the whole colon was on the left hemi-abdomen and the whole small bowel was on the right with the ligament of Trietz at the usual site of the right hepatic flexure situated just inferior to the right lobe of the liver. The appendix was on the left side with perforation at the middle third forming adhesion with greater omentum (Fig. 7 and Additional file 1). There was around 1 L of thin pus in the peritoneal cavity. All other intraabdominal organs were in their usual anatomic sites. For these findings the pus was drained, appendectomy done and the peritoneum was lavaged with warm normal saline, the fascia closed and the skin left open for wound care. Postoperatively, the patient was commenced on ceftriaxone 1 g IV twice a day, metronidazole 500 mg IV three times a day, and diclofenac 50 mg IV twice a day.

**Table 1:** Vital signs and physical examination finding of the patient at addmission, at 48 hours and at discharge

Parameters	At addmission	At 48 hours (immediately before operation)	At discharge
Pulse rate	84 bpm	100 bpm	72
Blood pressure	110/70 mmHg	100/70 mmHg	110/80 mmHg
Respiratory rate	14 bpm	16 bpm	12 bpm
Temperature	36.9 °C	37.7 °C	36.5 °C
Tenderness	Localized (left lower quadrant)	Diffuse	Minimal tenderness around the wound site

The patient was extubated safely and transferred to the recovery. The postoperative course was uneventful, and he was discharged improved on the 6th postoperative day. The patient was followed after discharge, and no complications were noted up to 3 weeks postoperative period.

## Discussion

Situs inversus (SI) and midgut malrotation (MM) are uncommon anatomic anomalies that complicate its diagnosis and management. MM is a rare congenital anomaly; it occurs due to an error in the process of rotation or fixation of intestines around the superior mesenteric vessels during embryologic development and it results in non-rotation or incomplete rotation of intestines with an abnormal location of the intestine or the appendix [7]. Left-sided acute appendicitis (LSAA) is a very rare cause of acute abdomen, developing in association with two types of congenital anomalies, situs inversus (SI) and midgut malrotation (MM). Appendicitis rarely presents with left lower abdominal pain especially when the intestine is non-rotated or mal-rotated. Its diagnosis becomes quite troublesome to clinicians and delays prompt intervention [8]. Intestinal malrotation is a rare clinical entity that occurs in 1/6000 live births and non-rotation is the most common type. The presence of undiagnosed congenital anomalies such as MM can render a diagnosis of even seemingly straightforward conditions such as acute appendicitis challenging [9].

Symptoms of MM are not well understood and it may remain asymptomatic in most cases. In the case of nonrotation, while the small intestines are observed on the right side, the large intestines are observed on the left side of the midline which is similar to the case of our patient [10]. Adult MM is rare and the majority of MM in adults remains asymptomatic throughout life. The increasing use of diagnostic imaging for acute abdominal pain leads to more incidental recognition of MM [11]. In our case, the patient had no history of previous chronic abdominal pain and this is his first presentation to a hospital. Preoperative clinical diagnosis of LSAA is very difficult and needs a high index of suspicion and imaging may help determine the correct diagnosis, as well as confirm SI or MM. LSAA is a rare surgical emergency that should be considered in the differential diagnosis of patients with left-side abdominal pain of atypical presentation [12]. The epigastric and left paraumbilical pain and the presence of acute watery diarrhea in our case made the presentation atypical.

Ultrasound and CT scans are the preferred methods of imaging modalities in the diagnosis of intestinal mal-rotation. The main features of intestinal mal-rotation include the absence of a retro mesenteric retroperitoneal segment of the duodenum, medially and inferiorly oriented duodenojejunal junction, abnormal inversion of SMA and SMV relationships, left side large bowel and predominantly right-side small bowel [13].

The surgeon should be informed about the embryology and anatomy of the intestinal rotation [4]. A delay in diagnosis will lead to a delay in definitive management. There was a delay in diagnosis of about 48 hours in our case. Even though both laparoscopic and open surgery has been used in the past [14], a laparoscopic approach is a useful and safe procedure both for the diagnosis and treatment of such unclear clinical presentations [7]. Clinicians should bear in mind the possibility of underlying midgut malrotation, as appendicitis could be the first presentation of this rare congenital condition [15]. Given the rarity of acute appendicitis associated with intestinal malrotation, an increase in awareness of this anatomical variant is essential among emergency physicians, radiologists, and surgeons for prompt diagnosis and timely intervention [16]. Left-side acute appendicitis is a very rare clinical condition and only a few case reports are published in the literature. As far as our knowledge is concerned, no case reports have been reported in Ethiopia so far.

## Conclusions

The rare presentation of left-side acute appendicitis poses a diagnostic challenge for surgeons and radiologists. Hence, it results in delays in patient diagnosis and management. An increase in awareness of this anatomical variant is essential among emergency physicians, radiologists, and surgeons for prompt diagnosis and timely intervention of this very rare clinical to minimize the delay of patient management and unnecessary perioperative complications.

## Supplementary Information

**Additional file 1.** Images added for further reference.

## Data Availability

The authors of this manuscript are willing to provide additional information regarding the case report.

## Abbreviations

AA: Acute appendicitis
CT: Computed tomography
GI: Gastro-intestinal
IV: Intravenous
LSAA: Left side acute appendicitis
MM: Mid-gut mal-rotation
NG tube: Naso-gastric tube
SI: Situs inversus
SMA: Superior mesenteric artery
SMV: Superior mesenteric vein
USG: Ultrasonography
