# Supplementary figures and images for: Left side perforated appendicitis with intestinal non-rotation: a case report
Source: J Med Case Rep. 2023 Jun 30;17:302. doi: 10.1186/s13256-023-03990-2 (PMC10311855; doi:10.1186/s13256-023-03990-2)

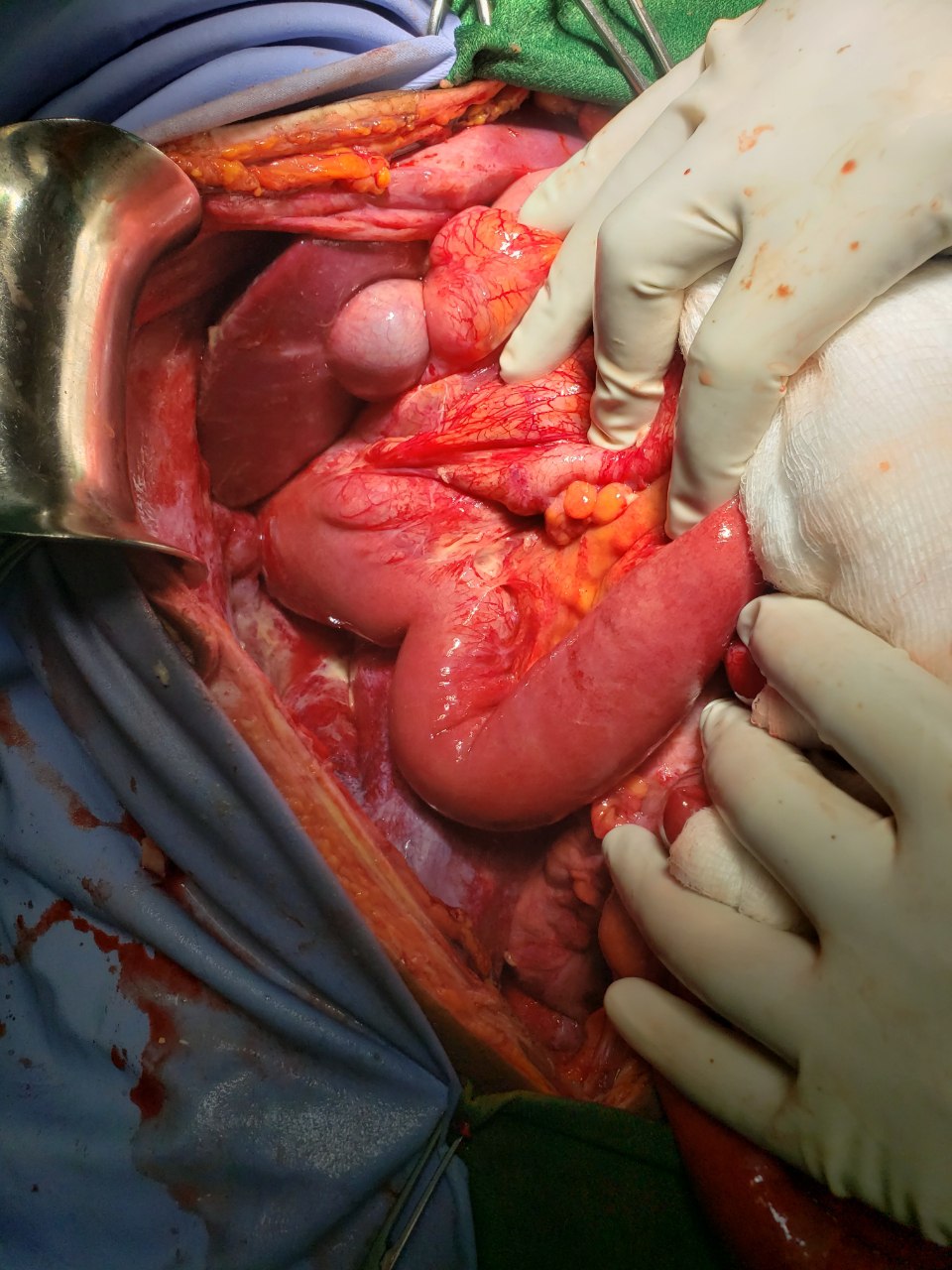

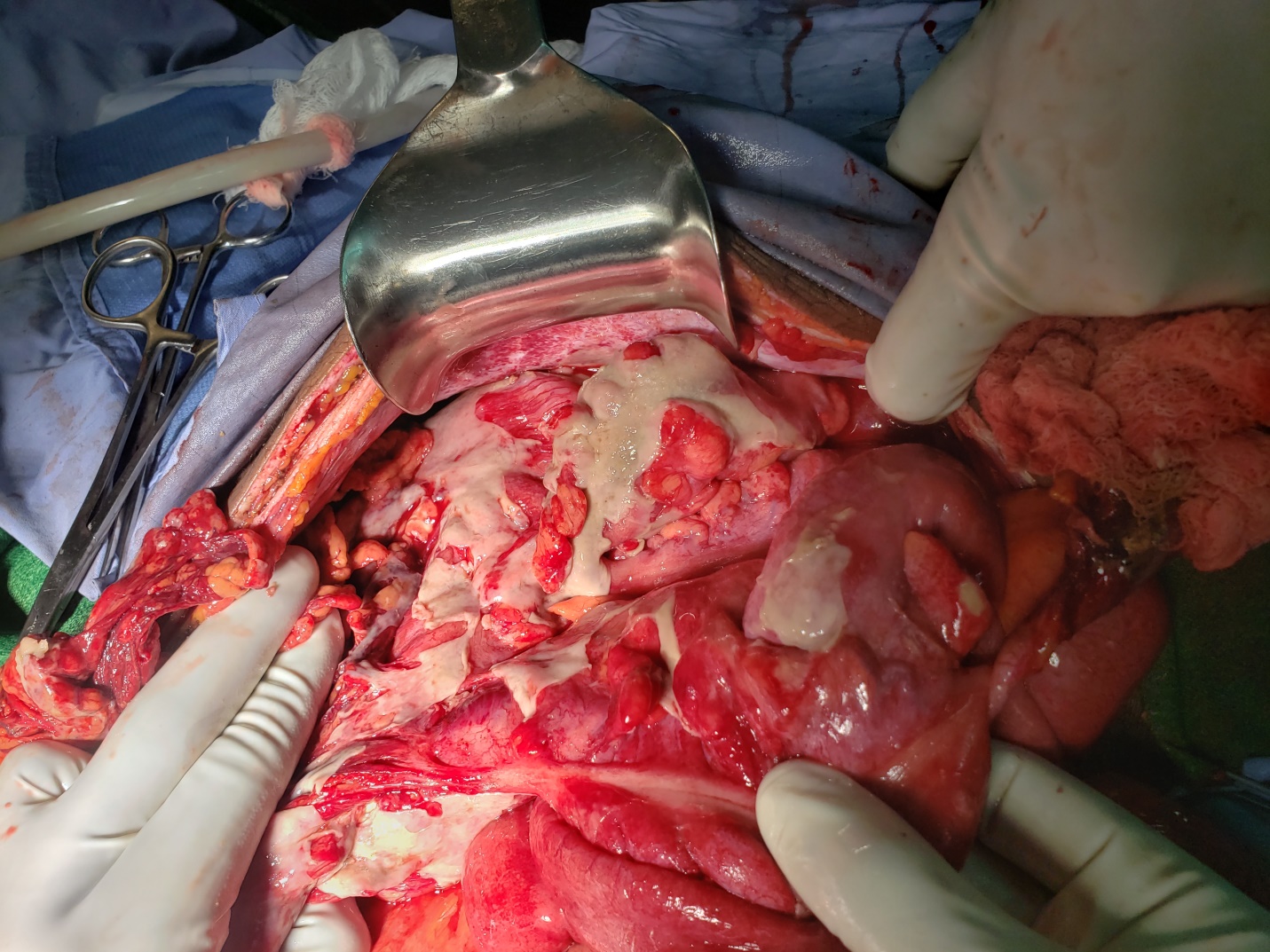


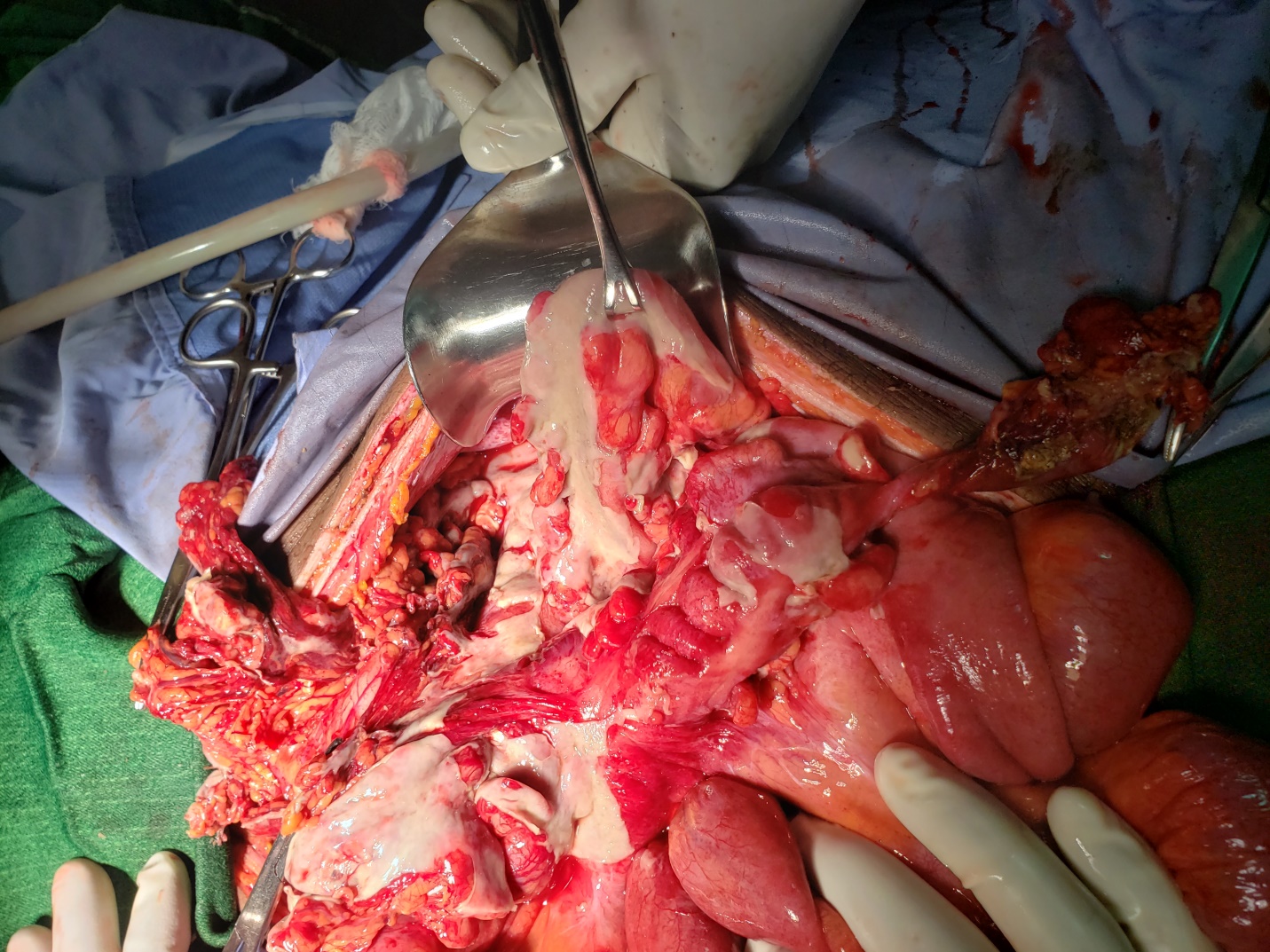


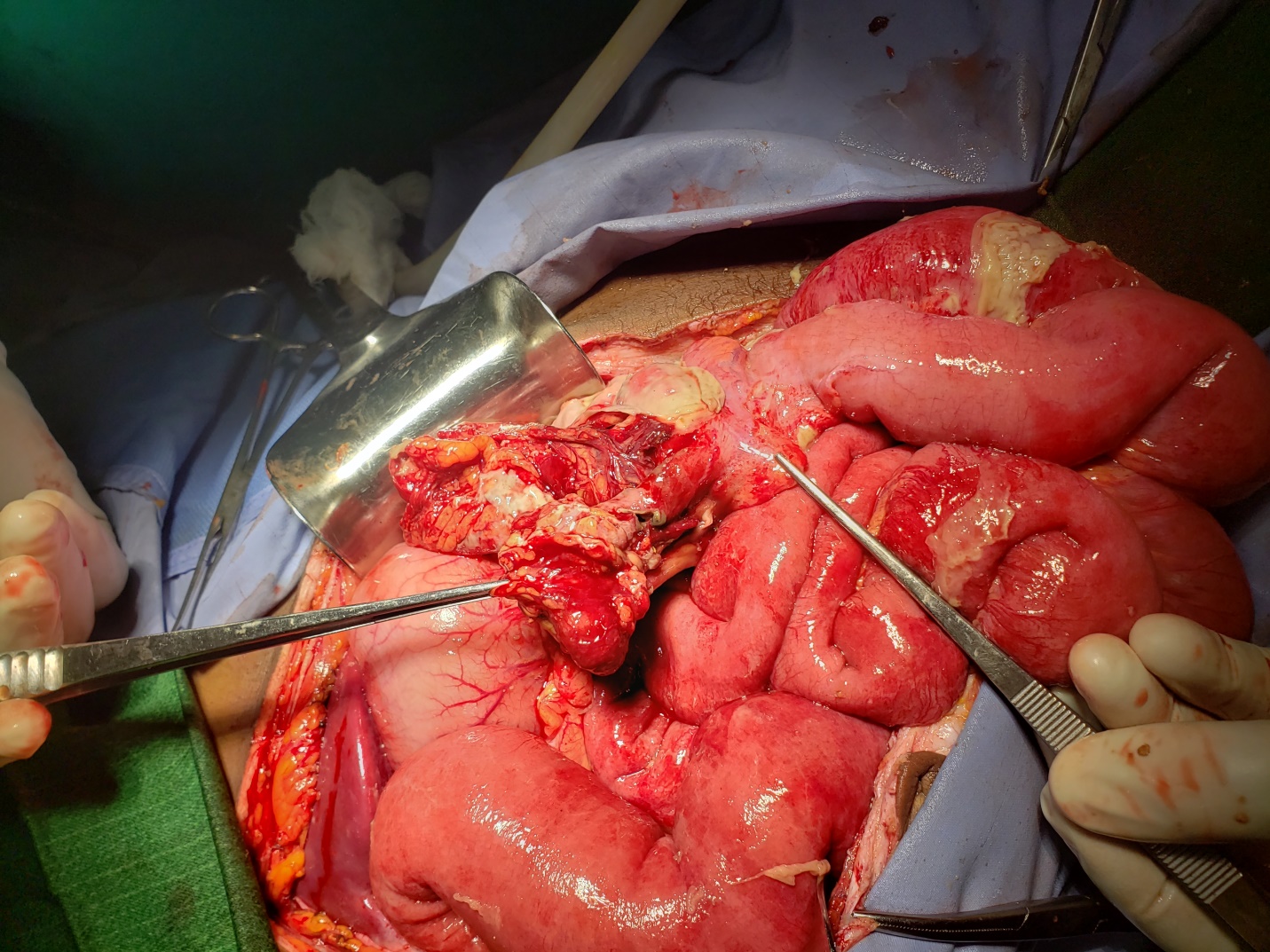


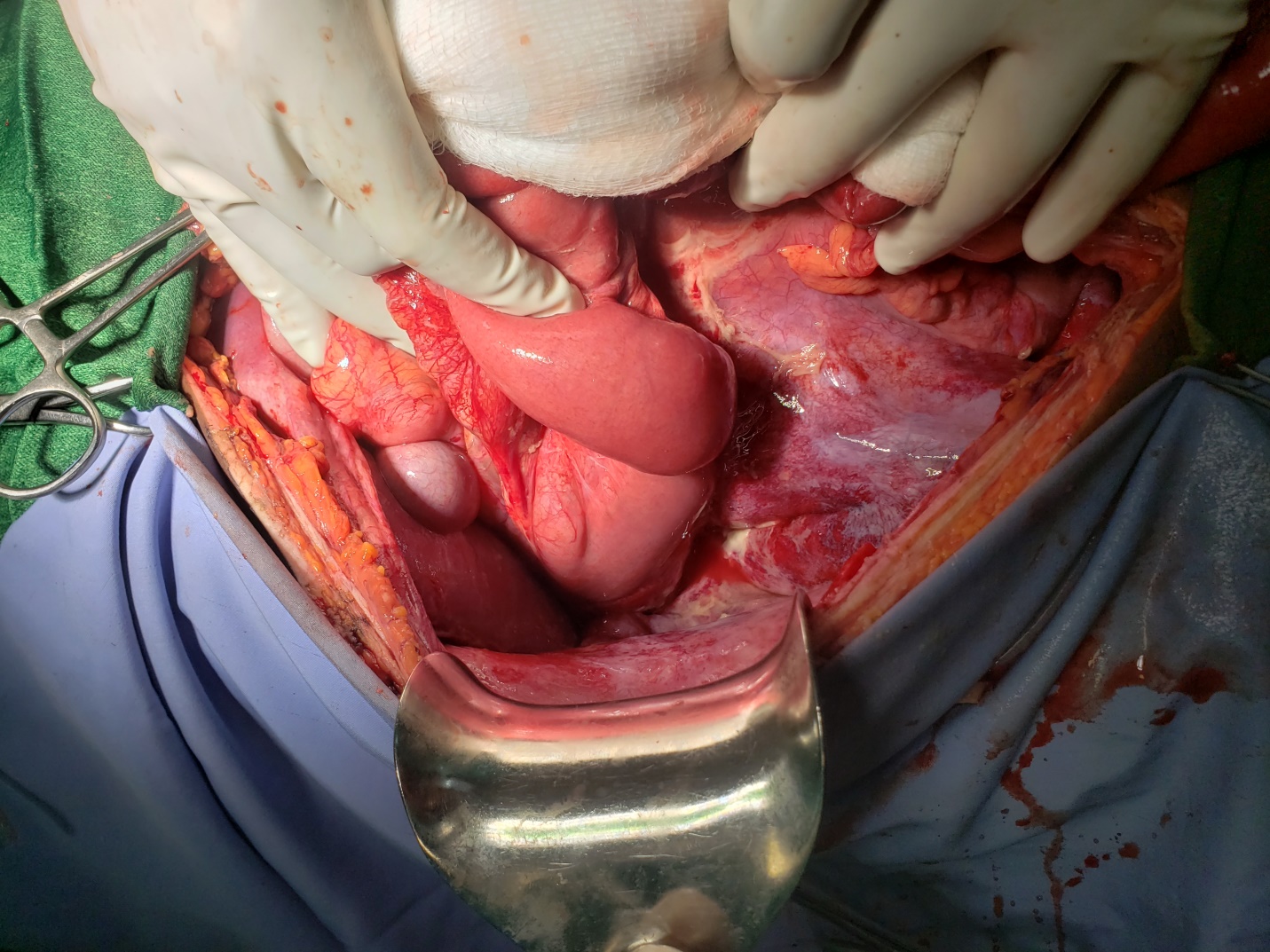


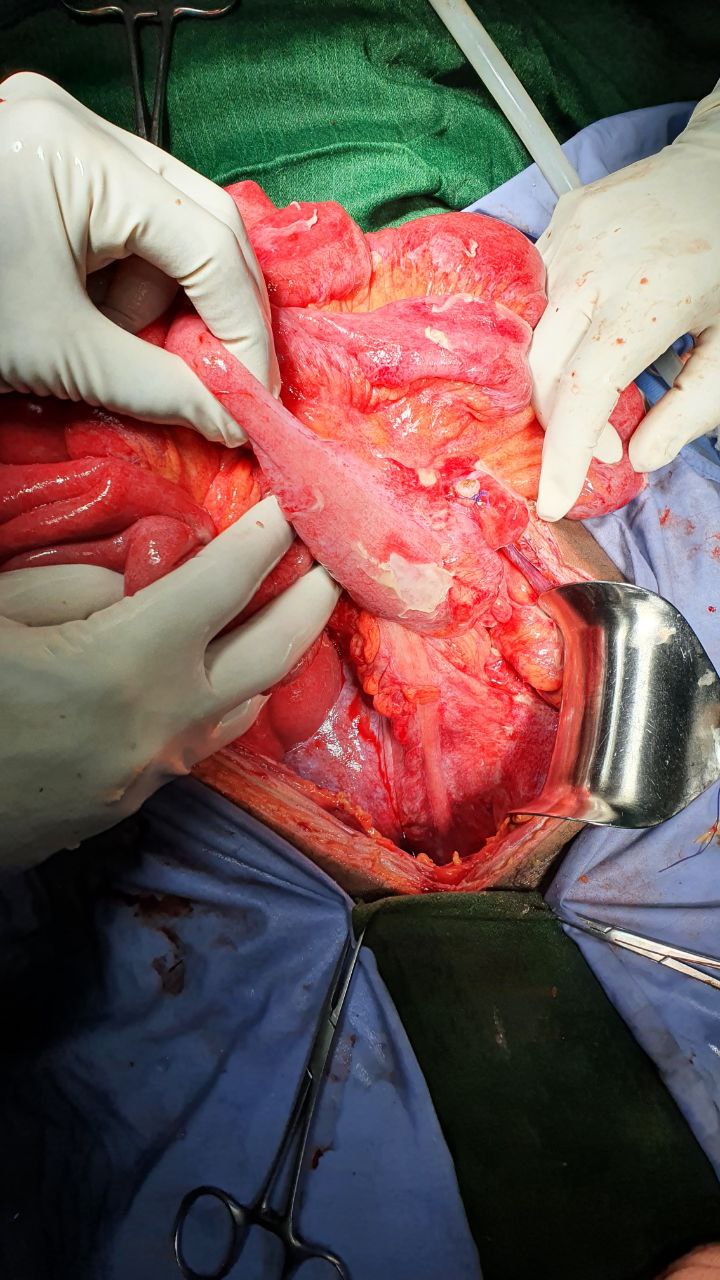


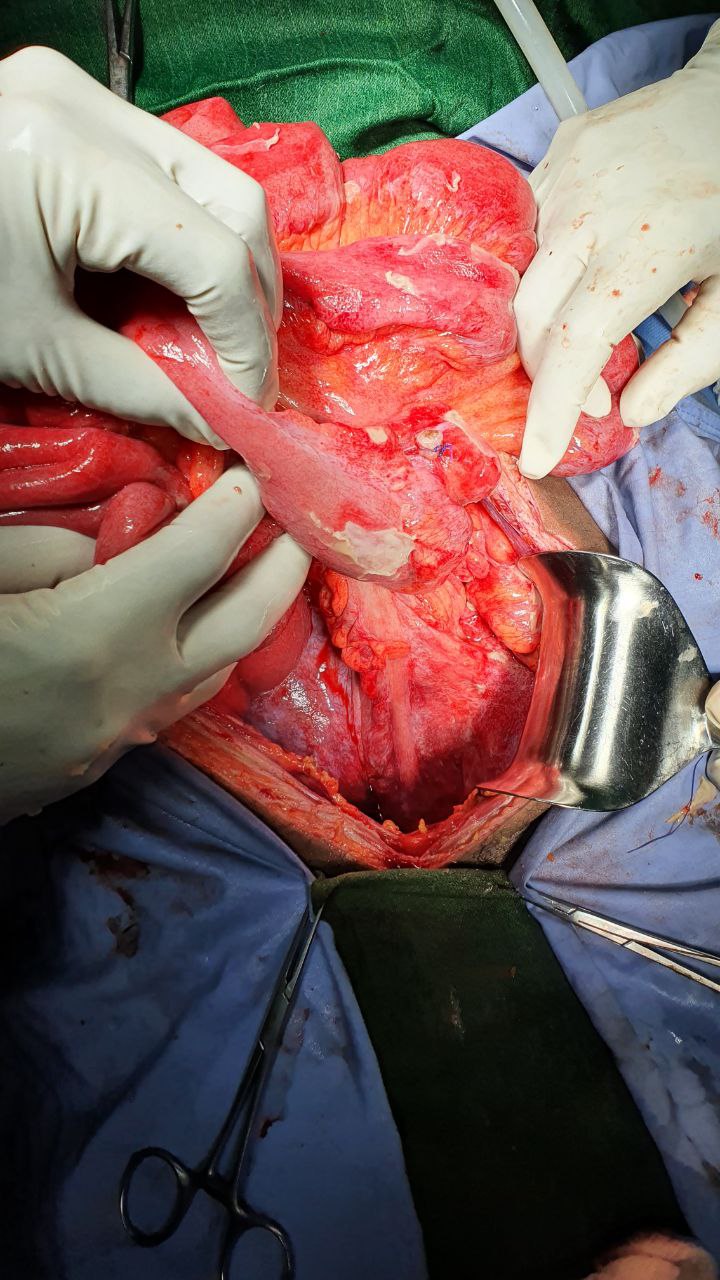


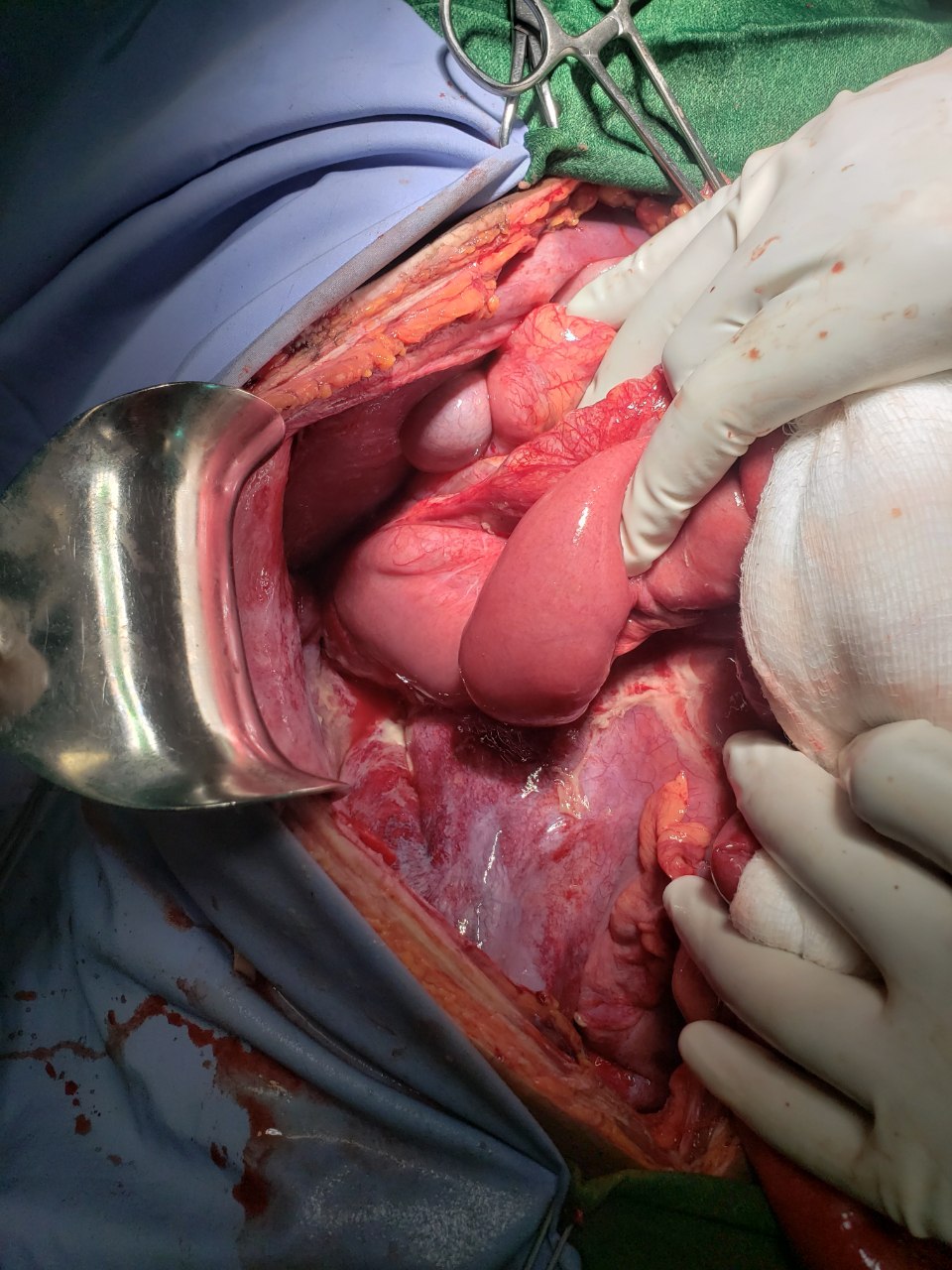


Additional file 1: images added for further reference

Supplement: Supplementary file 1 — Additional file 1. Images added for further reference. [file 13256_2023_3990_MOESM1_ESM.docx]
